# Computational data of molybdenum disulfide/graphene bilayer heterojunction under strain

**DOI:** 10.1016/j.dib.2022.108054

**Published:** 2022-03-13

**Authors:** Nicholas Dimakis, Sanju Gupta, Razeen Wadud, Muhammad I. Bhatti

**Affiliations:** a Department of Physics and Astronomy, University of Texas Rio Grande Valley, Edinburg 78539, USA; b Department of Materials Science and Engineering, Pennsylvania State University, University Park 16802, USA

**Keywords:** DFT, Heterostructures, QTAIM, NCI

## Abstract

The data presented in this paper refer to the research article “Dry and Hydrated Defective Molybdenum Disulfide/Graphene Bilayer Heterojunction Under Strain for Hydrogen Evolution from Water Splitting: A First-principle Study”. Here, we present the Density Functional Theory (DFT) data used to generate optimal geometries and electronic structure for the MoS_2_/graphene heterostructure under strain, for dry and hydrated pristine and defect configurations. We also report DFT data used to obtain hydrogen Gibbs free energies for adsorption on the MoS_2_ monolayer and on graphene of the heterostructure. The DFT data were calculated using the periodic DFT code CRYSTAL17, which employs Gaussian basis functions, under the hybrid functionals PBE0 and HSE06. Moreover, we also report the data used for Quantum Theory of Atoms in Molecules (QTAIM) and Non-covalent Interaction (NCI) analysis calculations. These data were obtained using the optimized unit cell configurations from the periodic DFT and inputted to Gamess program, thus generating files that could be read by the Multiwfn program used for QTAIM and NCI calculations.

## Specifications Table

**Table:** 

Subject	*Chemistry*
Specific subject area	*Computational Chemistry*
Type of data	Table Figure
How the data were acquired	*Optimized geometries and electronic structure calculations were obtained using the CRYSTAL17 program. QTAIM and NCI calculations were obtained the using the Multiwfn program. The unit cell geometries obtained from the CRYSTAL17 program were inputted to the Gamess program to produce output files read by the Multiwfn program.*
Data format	Raw Analyzed
Description of data collection	Computational DFT data were obtained using CRYSTAL17, Gamess, and Multiwfn programs running at using the Texas Advanced Computing Center (TACC)Canter Ce Center facilities. Band structure output data (extension *f25) are read by CRYSPLOT (https://crysplot.crystalsolutions.eu).
Data source location	• Institution: University of Texas Rio Grande Valley • City/Town/Region: Edinburg, TX • Country: USA
Data accessibility	*Within the article and under Mendeley Data* Repository name: Mendeley Data Data identification number: 10.17632/dxgvy7mzrn.1 Direct URL to data: https://data.mendeley.com/datasets/dxgvy7mzrn/1
Related research article	*N. Dimakis, S. Gupta, R. Wazzen, M. I. Bhatti, Dry and Hydrated Defective Molybdenum Disulfide/Graphene Bilayer Heterojunction Under Strain for Hydrogen Evolution from Water Splitting: A First-principle Study, Comput. Mat. Sci. 205 (2022) 111234*[1]. https://doi.org/10.1016/j.commatsci.2022.111234

## Value of the Data

• We provide (a) structural and electronic information for dry and hydrated pristine and defect MoS_2_/graphene as calculated by density functional theory (DFT) and (b) outputs from quantum theory of atoms in molecules (QTAIM) and Non-covalent Interaction (NCI) calculations. Defect MoS_2_/graphene heterostructures serve as hydrogen evolution catalysts (HER).
• Electronic information shows a bandgap opening at the Dirac point region, which is affected by hydration and vacancies. Thus, this bandgap could be engineered for producing efficient HER electrocatalysts.
• The presence of QTAIM S-C bond critical points and NCI calculations show that MoS_2_-graphene interaction is var der Waals.
• We also provide data that support the MoS_2_/graphene use as HER, when S and C defects are included in the lattice. These data can be further used by experimentalists to examine the needed concentration of S that produce an HER catalyst with hydrogen Gibbs energy of approximately zero.

## 1 Data Description

**Fig 1:**
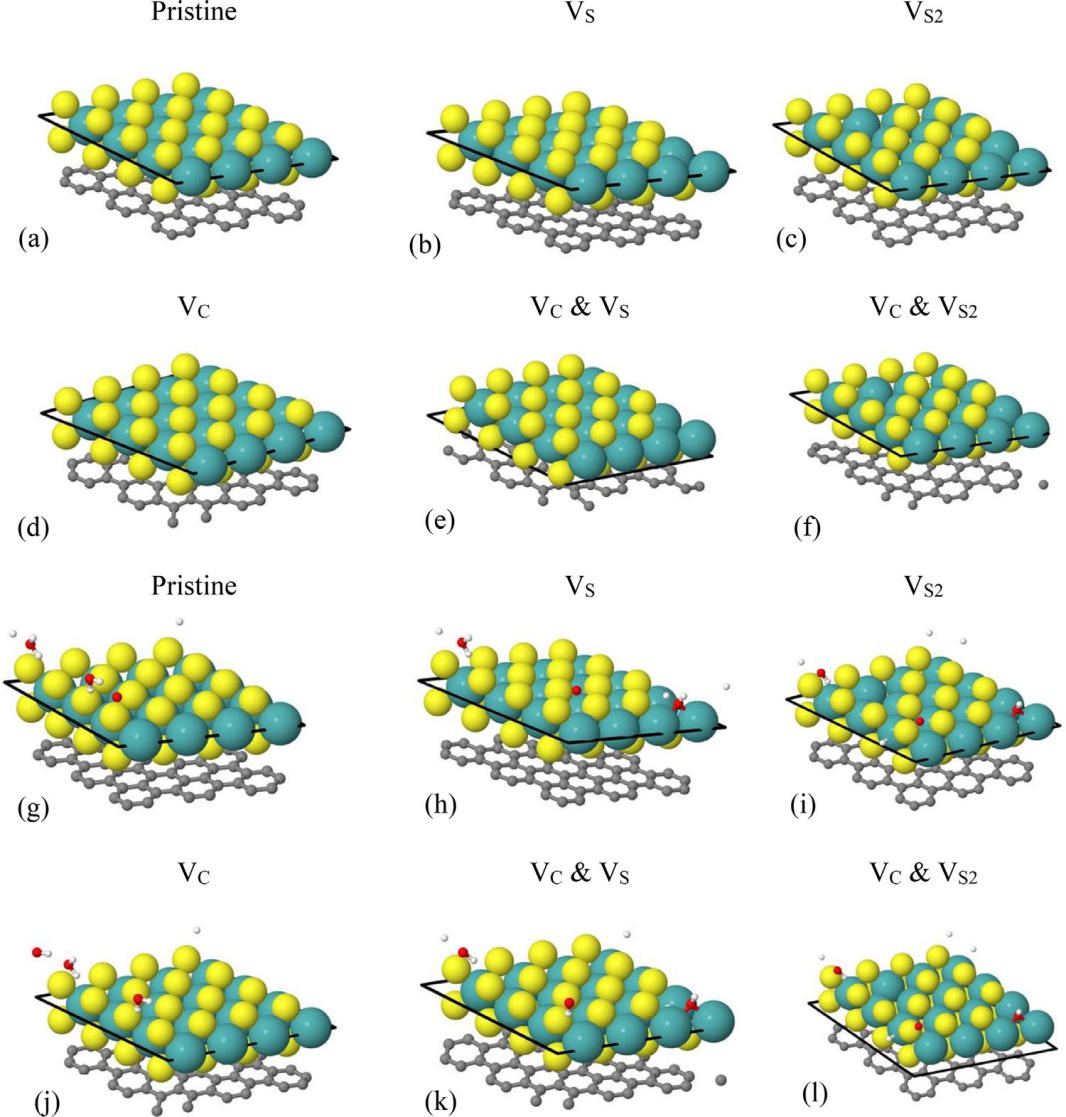
DFT optimized unit cells for MoS_2_/graphene using the PBE0 functional under the following configurations: (a) Dry pristine, (b)–(f) dry defect, (g) hydrated pristine, and (h)–(l) hydrated defect. The thick black lines are the unit cell boundaries. The S, Mo, C, H, and O atoms are shown in yellow, green, gray, white, and red, respectively. Visualization is via Jmol.

**Fig. 2:**
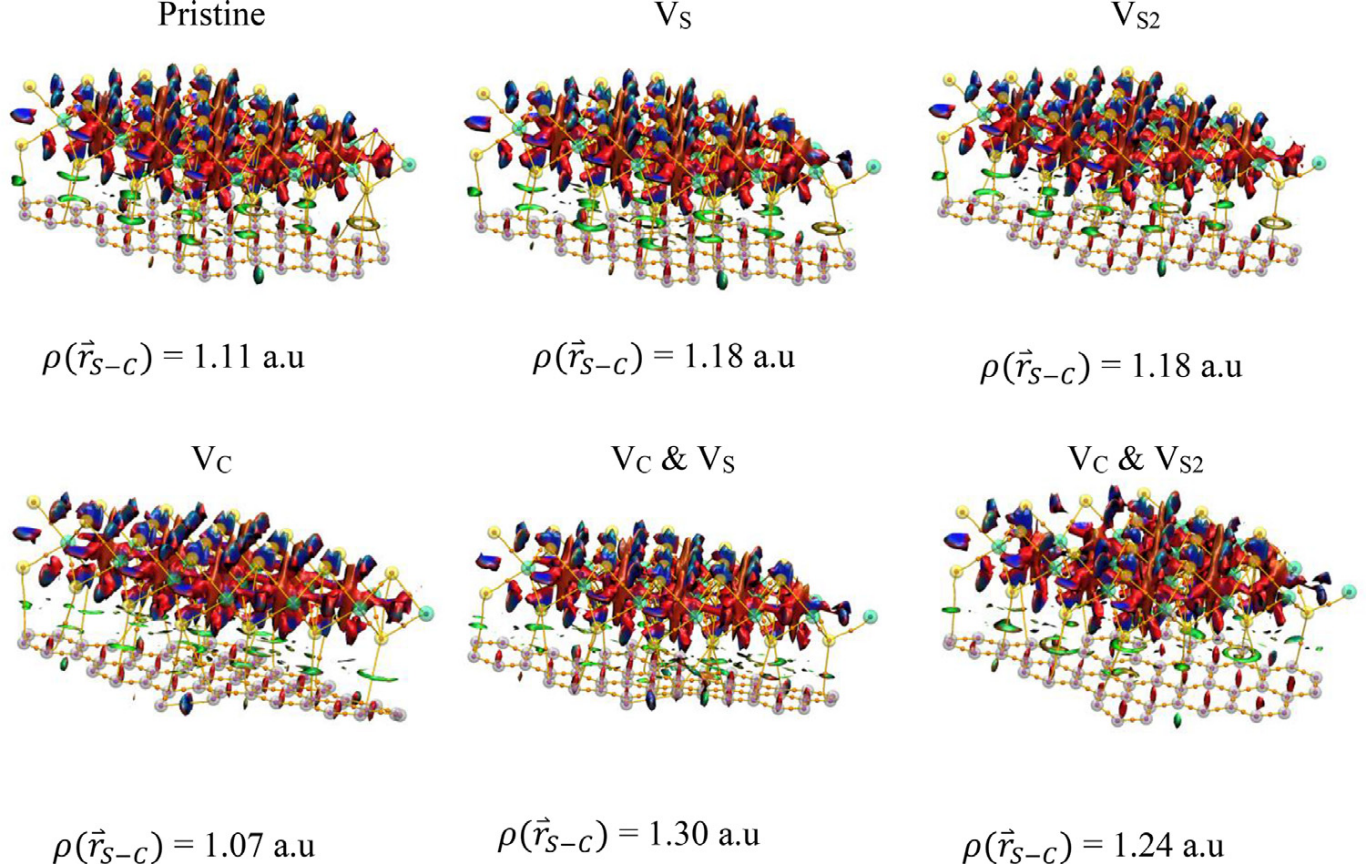
QTAIM molecular graphs and NCI isosurfaces obtained from the DFT optimized unit cell for dry MoS_2_/graphene using Multiwfn and plotted via VMD [2]. Small and large spheres denote critical points and atoms, respectively. Atoms colors areas follows: S, yellow; Mo, green; C, carbon. QTAIM critical points are colors as follows: nuclear critical points, purple; bond critical points, orange. Surface colors are as follows: Green: van den Waals; red, repulsion; blue, attraction. The $\rho ({\vec{r}}_{S-C})$ values reported are average values for the entire unit cell.

Fig. 1 shows the optimal geometries for dry and defect MoS_2_/graphene heterostructures with and without interacting waters on the S surface of the MoS_2_, as calculated by the PBE0 functional. We found no significant differences in the above optimal geometries for calculations using the HSE06 functional. Fig. 1(g)–(l) show that one of the three waters in the unit cell dissociates at the S vacancy region. Table 1 shows the Dirac point locations (E_D_), minigaps (ΔE), and MoS_2_ bandgaps (E_g_) for pristine and defect configurations of this work under the HSE06 calculations. Corresponding E_D_, ΔE, and E_g_ using the PBE0 functional are shown on Table 2. This information is produced by electronic band structure calculations. The PBE0 cal overestimate the MoS_2_ bandgaps E_g_ relative to the HSE06 calculations, as expected. Table 3 shows the Gibbs free energy ΔG_H_ for H adsorption on MoS_2_ and graphene for each MoS_2_/graphene configuration under the PBE0 calculations. For H/MoS_2_, ΔG_H_ is positive for adsorption on pristine MoS_2_ and negative, when S vacancies are present. Fig. 2 shows QTAIM molecular graphs and NCI isosurfaces were obtained from the DFT optimized unit cell geometries for dry MoS_2_/graphene using Multiwfn and plotted via VMD (PBE0 calculations). Thus, for these calculations a molecular cluster has been used.

**Table 1:** Approximate Dirac point locations (E_D_), minigaps (ΔE), and MoS_2_ bandgaps (E_g_) for pristine and defect configurations of this work under the HSE06 calculations. Values in parenthesis refer to the hydrated cases.

MoS_2_/Graphene	E_D_*(eV)*	*ΔE (meV)*	*E_g_ (eV)*
Pristine	*0.22*	*0.05*	*1.74*
	*(0.22)*	*(0.90)*	*(1.76)*
V_S_	*0.44*	*7.29*	*1.07*
	*(0.25)*	*(14.20)*	*(1.67)*
V_S2_	*0.51*	*2.04*	*0.42*
	*(0.39)*	*(13.29)*	*(0.74)*
V_C_	*0.24*	*46.94*	*1.93*
	*(0.23)*	*(47.37)*	*(1.94)*
V_C_ & V_S_	*0.37*	*66.51*	*1.18*
	*(0.24)*	*(20.35)*	*(1.63)*
V_C_ & V_S2_	*0.44*	*79.6*	*0.59*
	*(0.30)*	*(57.39)*	*(0.79)*

**Table 2:** Approximate Dirac point locations (E_D_), minigaps (ΔE), and MoS_2_ bandgaps (E_g_) for pristine and defect configurations of this work under the PBE0 calculations. Values in parenthesis refer to the hydrated cases.

MoS_2_/Graphene	E_D_*(eV)*	*ΔE (meV)*	*E_g_ (eV)*
Pristine	*0.14*	*0.44*	*2.33*
	*(0.13)*	*(0.90)*	*(2.36)*
V_S_	*0.39*	*19.24*	*1.64*
	*(0.16)*	*(15.56)*	*(2.27)*
V_S2_	*0.50*	*5.91*	*0.80*
	*(0.34)*	*(21.39)*	*(1.32)*
V_C_	*0.15*	*6.86*	*2.54*
	*(0.15)*	*(10.00)*	*(2.55)*
V_C_ & V_S_	*0.30*	*61.30*	*1.74*
	*(0.25)*	*(53.23)*	*(2.27)*
V_C_ & V_S2_	*0.50*	*74.12*	*1.01*
	*(0.33)*	*(11.15)*	*(1.31)*

**Table 3:** Gibbs free energy (ΔG_H_) for H adsorbed on MoS_2_ and graphene for each MoS_2_/graphene configuration under the PBE0 calculations.

MoS_2_/Graphene	$\Delta G_{H}^{H/\text{Mo}S_{2}}\left(\text{eV}\right)$	$\Delta G_{H}^{H/\text{Graphene}}\left(\text{eV}\right)$
Pristine	1.89	0.83
V_S_	−1.70	0.88
V_S2_	−1.82	0.85
V_C_	1.94	−2.57
V_C_ & V_S_	−1.67	−0.52
V_C_ & V_S2_	−1.77	−2.57

The submitted data are grouped in five directories. Two directories contain CRYSTAL17 input and output files from electronic band structure and densities of states (DOS) calculations and from optimized geometries. Each of these directories contain information from HSE06 and PBE0 calculations, which are found in separate subdirectories. The other three directories contain HER calculations for the PBE0 functional (CRYSTAL 17 input and output files), Gamess input and output files, and QTAIM-NCI Multiwfn output files.

## 2 Experimental Design, Materials and Methods

We constructed the pristine dry MoS_2_/graphene heterostructure under comprehensive strain by using a 2H-MoS_2_ three-layer 4 × 4 supercell of 48 atoms overlayered on a 3$\sqrt{3}\times$ 3$\sqrt{3}\;R30^{\circ }$ supercell monolayer graphene. This configuration contains 32 S, 16 Mo, and 54 C atoms totaling 102 atoms. Hydrated MoS_2_ surfaces contain three water molecules per unit cell. These waters are placed on the S layer away from graphene. We built defective MoS_2_/graphene heterostructures by considering all combinations of single (V_S_) and non-adjacent double S vacancies (V_S2_) in the MoS_2_ with a single C vacancy (V_C_) in the graphene layer.

Optimized geometries and electronic structure of all MoS_2_/graphene configurations were obtained using the periodic DFT code CRYSTAL17 [3], which employs Gaussian basis functions centered at the atoms. We used two DFT hybrid functionals for our calculations: The PBE0 non-empirical/parameter-free functional [4,5] and the HSE06 screened hybrid functional of Heyd, Scuseria, and Ernzerhof. HSE06 provides band gaps in better agreement with experimental findings [6]. Long-range electron correlations responsible for van der Waals interactions were treated by the Grimme D3 semiempirical correction [7]. The S, C, O, and H atoms are described by all-electron basis sets optimized for crystalline calculations. Specifically, the triple-zeta valence with polarization (TZVP) functions were used for the S atoms as 73211/5111/1 for s/p/d functions, where 73211 stands for 7, 3, 2, and 1 contracted Gaussians to describe the 1s shell, 2s, 3s, and 4s shells, respectively. Moreover, the C and H atoms were described as 6211/411/1 for s/p/d functions and 311/1 for s/p functions, respectively, whereas the O atoms used the split-valence basis set 8-411G(2d1f), where the 6 electrons on the 2s and 2p shells were described by 4 sp functions. Mo atoms use effective core potentials (ECP) and double zeta basis set with polarization for its valence as 311/41/41/1 for s/p/d functions [8]. Geometry optimizations were obtained using a 6 × 6 Monkhorst-Pack, whereas the electronic band structures calculations and DOS used a 24 × 24 grid. Band structure calculations used the following path M-Γ-Κ-M-Γ, with M (1/2, 0, 0), Γ (0, 0, 0), and Κ (1/3, 1/3, 0). The Γ point of the supercell Brillouin zone (BZ) coincides with the K point of the unit cell BZ. Thus, the E_D_ appears at the Γ point.

Hydrogen adsorption energies $E_{ads}\left(H\right)$ are calculated by

$$E_{ads}\left(H\right)=\;E\left(\text{Mo}S_{2}/\text{Graphene}-H\right)-E\left(\text{Mo}S_{2}/\text{Graphene}\right)-\frac{1}{2}E\left(H_{2}\right)$$

where $E(\text{Mo}S_{2}/\text{Graphene}-H)$ and $E(\text{Mo}S_{2}/\text{Graphene})$ are the total energies for the heterostructure with and without adsorbed H, respectively and $E(H_{2})$ is the energy of the free H_2_. The Gibbs free energy $\Delta G_{H}$ for the adsorbed hydrogen is given by

$$\Delta G_{H}=\;E_{ads}\left(H\right)+\Delta G_{ZPE}-T\Delta S_{H}$$

where $\Delta G_{ZPE}$ and $\Delta S_{H}$ are the zero-point energy (ZPE) and entropy difference between the adsorbed and the gas phase states, respectively. $\Delta G_{H}$ values are approximated as

$$\Delta G_{H}≅\;E_{ads}\left(H\right)+0.24\;\text{eV}$$

QTAIM [9,10] and NCI [11] information were obtained using the Multiwfn [12] program. This program does not accept information directly from CRYSTAL17 output files. For this reason, we extracted a cluster from the periodic layer from the CRYSTAL17 output files, which corresponds to the optimal geometry per configuration, and generated Gamess [13] input files for single energy calculations. These outputs from Gamess, served as inputs to Multiwfn. QTAIM calculations provided the electron density ($\rho (\vec{r})$) and its Laplacian ($\nabla^{2}\rho \left(\vec{r}\right)$) at all bond critical points. Since QTAIM analyses are basis set and method independent, [14] we only used the PBE0 functional here. Weak interactions were studied via the NCI method, by calculating the reduced density gradient (RDG) parameter ($\propto {}^{|\nabla^{2}\rho \left(\vec{r}\right)|}/{}_{\rho {\left(\vec{r}\right)}^{4/3}}$) and plotting the RDG map.

## Ethics Statements

None.

## CRediT authorship contribution statement

**Nicholas Dimakis:** Conceptualization, Methodology, Supervision, Validation, Writing – review & editing. **Sanju Gupta:** Conceptualization, Methodology, Writing – review & editing. **Razeen Wadud:** Visualization, Investigation, Validation. **Muhammad I. Bhatti:** Writing – review & editing.

## Declaration of Competing Interest

The authors declare that they have no known competing financial interests or personal relationships that could have appeared to influence the work reported in this paper.

The authors declare the following financial interests/personal relationships which may be considered as potential competing interests:

## References

[1] Dimakis N., Gupta S., Wazzen R., Bhatti M.I. (2022). Dry and hydrated defective molybdenum disulfide/graphene bilayer heterojunction under strain for hydrogen evolution from water splitting: a first-principle study. Comput. Mater. Sci.

[2] Humphrey W., Dalke A., Schulten K. (1996). VMD - visual molecular dynamics. J. Mol. Graph..

[3] R. Dovesi, V.R. Saunders, C. Roetti, R. Orlando, C.M. Zicovich-Wilson, F. Pascale, B. Civalleri, K. Doll, N.M. Harrison, I.J. Bush, P. D'Arco, M. Llunell, M. Causà, Y. Noël, L. Maschio, A. Erba, M. Rérat, S. Casassa, CRYSTAL17 user's manual (University of Torino, Torino, 2017).

[4] Ernzerhof M., Scuseria G.E. (1999). Assessment of the Perdew–Burke–Ernzerhof exchange-correlation functional. J. Chem. Phys..

[5] Adamo C., Barone V. (1999). Toward reliable density functional methods without adjustable parameters: the PBE0 model. J. Chem. Phys..

[6] Krukau A.V., Vydrov O.A., Izmaylov A.F., Scuseria G.E. (2006). Influence of the exchange screening parameter on the performance of screened hybrid functionals. J. Chem. Phys..

[7] Grimme S., Antony J., Ehrlich S., Krieg H. (2010). A consistent and accurate ab initio parametrization of density functional dispersion correction (DFT-D) for the 94 elements H-Pu. J. Chem. Phys..

[8] Laun J., Oliveira D.V., Bredow T. (2018). Consistent gaussian basis sets of double- and triple-zeta valence with polarization quality of the fifth period for solid-state calculations. J. Comput. Chem..

[9] Bader R.F.W. (1998). Encyclopedia of Computational Chemistry.

[10] Bader R. (1990).

[11] Johnson E.R., Keinan S., Mori-Sánchez P., Contreras-García J., Cohen A.J., Yang W. (2010). Revealing noncovalent interactions. J. Am. Chem. Soc..

[12] Lu T., Chen F. (2012). Multiwfn: a multifunctional wavefunction analyzer. J. Comput. Chem..

[13] Schmidt M.W., Baldridge K.K., Boatz J.A., Elbert S.T., Gordon M.S., Jensen J.H., Koseki S., Matsunaga N., Nguyen K.A., Su S., Windus T.L., Dupuis M., Montgomery J.A. (1993). General atomic and molecular electronic structure system. J. Comput. Chem..

[14] Jabłoński M., Palusiak M. (2010). Basis set and method dependence in atoms in molecules calculations. J. Phys. Chem. A.

